# Metachronous Metastatic Renal Cell Carcinoma (RCC) to the Urinary Bladder: A Rare Cause of Hematuria

**DOI:** 10.7759/cureus.82023

**Published:** 2025-04-10

**Authors:** Abdullah M Asiri, Mohamed A Rafie, Mohamed M Alalawi, Umar S Farouqi, Khalid M Alguthayr, Atheer Aboud, Adeel Khan, Shahista R Khan, Eman Aljuffairi, Fatima AlHashimi

**Affiliations:** 1 Training, King Hamad University Hospital, Busaiteen, BHR; 2 Urology, King Hamad University Hospital, Busaiteen, BHR; 3 College of Medicine and Medical Sciences, Arabian Gulf University, Manama, BHR; 4 Urology, Heartlands Hospital, University Hospitals Birmingham NHS Foundation Trust, Birmingham, GBR; 5 Radiology, King Hamad University Hospital, Busaiteen, BHR; 6 Pathology, King Hamad University Hospital, Busaiteen, BHR

**Keywords:** hematuria, metachronous, metastatic renal cell carcinoma, nephrectomy, urinary bladder

## Abstract

Renal cell carcinoma (RCC) metastasis to the urinary bladder has been a rarely reported clinical entity, and its potential metachronous pathobiological process poses significant challenges in RCC therapy-transforming scenarios. In this case, we share the experience of a female patient who underwent radical nephrectomy in September 2020 and was on regular follow-up. In March 2022, she was found to have right pulmonary metastasis on PET-CT imaging and accordingly underwent right-sided wide local excision of the lung lesion by thoracoscopy in June 2022, which revealed metastatic clear cell RCC. She was kept on immunotherapy as discussed in the tumor board meeting and was followed up. Uncommonly, in November 2022, a bladder growth was found involving the left vesicoureteric junction on follow-up CT scan, due to hematuria occurring two years after nephrectomy. Histopathologic examination revealed this mass to be an RCC metastasis. The subsequent imaging diagnosis led to an aggressive treatment approach, resulting in cystectomy with anterior exenteration and ileal conduit. This case is unique in Bahraini literature, as it highlights the unpredictable patterns of RCC metastasis and the infrequent occurrence of the bladder as the site of such metastasis. This report attempts to dissect the intricacies of RCC metastases to the bladder by providing an accurate perspective on diagnosing and managing such atypical sites through a literature review and in-depth discussion of histopathological characteristics. It highlights the importance of an aggressive follow-up regimen for RCC patients, potentially irrespective of their primary site appearing well contained. It would add to our understanding of a broader spectrum of RCC metastatic disease.

## Introduction

Metastatic renal cell carcinoma (RCC) of the urinary bladder is uncommon. The prognosis of patients with RCC metastasizing to the urinary bladder is poor [1]. RCC accounts for 2% of global cancer diagnoses, and about 33% of patients have metastatic disease at the time of diagnosis [2,3]. Lung (75%), liver (40%), bone (40%), soft tissues (34%), and pleura (31%) are the most common sites of RCC metastasis [4]. However, RCC can metastasize to almost any organ within the body, including the thyroid, pancreas, spleen, skin, intestine, heart, and urinary bladder [5].

About 40 cases have been reported, including the most extensive series of 11 patients with solitary metastases to the bladder [6]. We report a case of RCC with metachronous metastasis to the urinary bladder involving the contralateral ureteric orifice, occurring two years after radical nephrectomy. To our knowledge, this is the first case reported from Bahrain of RCC metastasis to the urinary bladder.

## Case presentation

**Figure 1:**
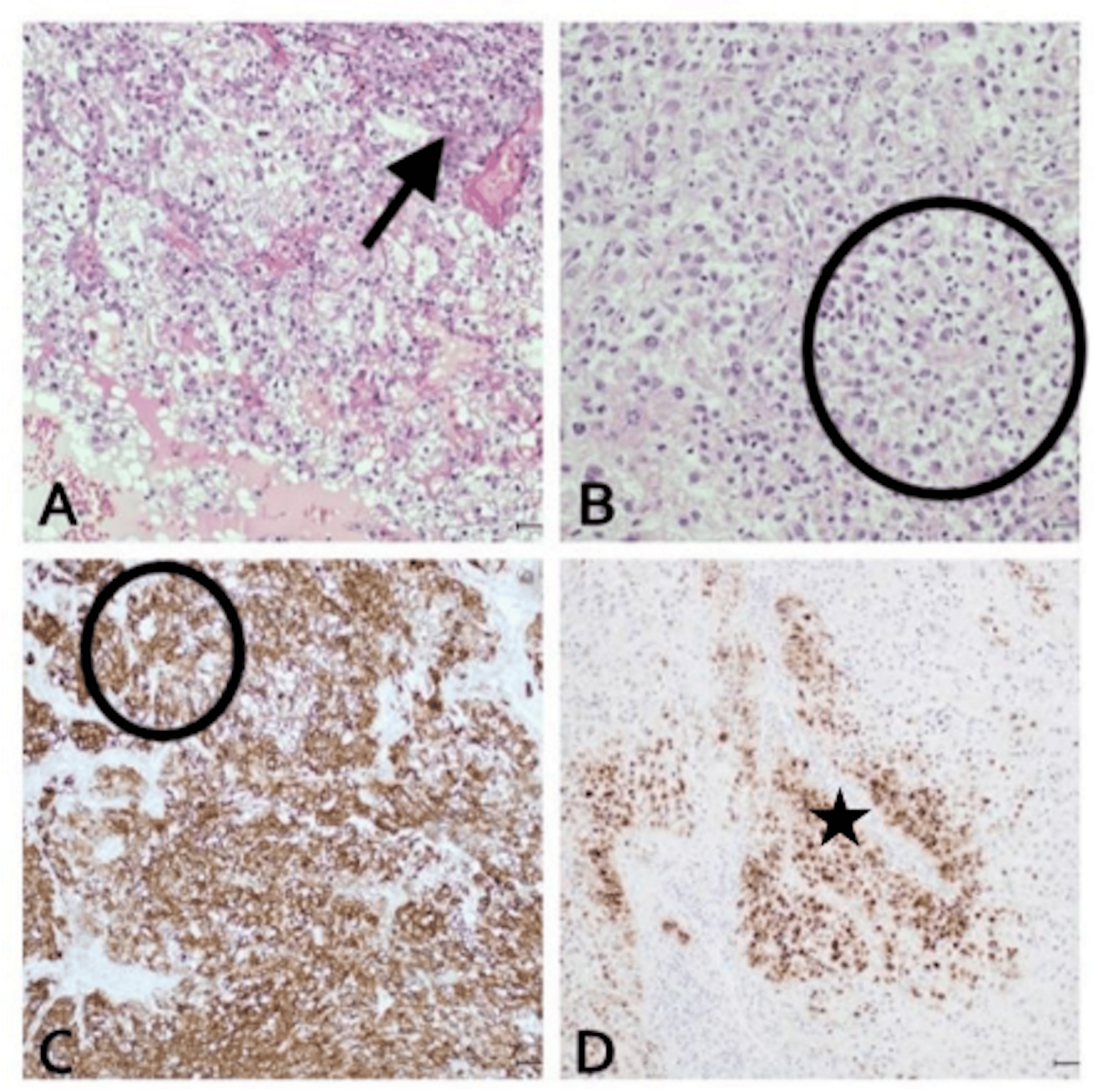
The nephrectomy specimen shows grade 4 clear cell renal cell carcinoma (A) with patchy rhabdoid differentiation (B). Immunohistochemical studies show that the tumor is positive for CD10 (C) and RCC marker (D).

A 64-year-old female with a history of diabetes mellitus, hypertension, and dyslipidemia, well controlled on medication, initially presented with a complaint of hematuria. On further evaluation, a triple-phase contrast CT of the abdomen was performed, which revealed a renal mass. The tumor measured approximately 10 cm and had a tumor thrombus in the right renal vein. The lesion was solid with central necrosis. She underwent a right radical nephrectomy with inferior vena caval thrombectomy and tumor extraction in September 2020. She had no known family history of urologic malignancy, and there were no laboratory abnormalities or signs and symptoms suggestive of paraneoplastic syndromes. Histopathology revealed renal cell carcinoma, grade 3 or 4, with tumor thrombus in the renal vein and focal invasion into the vascular wall. Resection margins were uninvolved, and no lymph node dissection was performed. The final pathologic stage was pT3c (Figure 1).

**Figure 2:**
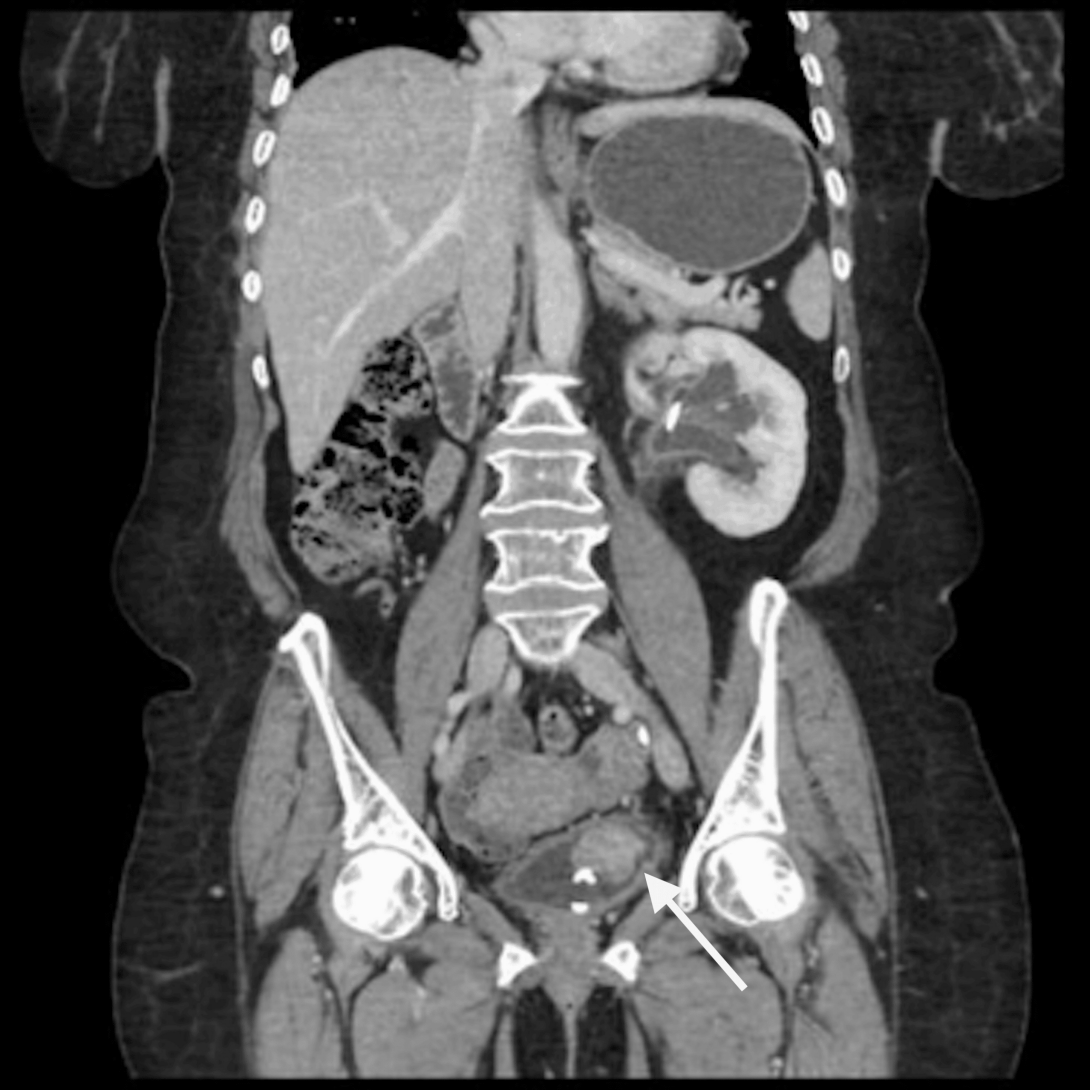
CT urography shows an enhancing mass arising near the left ureteric orifice with a DJ stent in situ.

The patient was advised to follow up as per the protocol based on the European Association of Urology and the EAU guideline for follow-up of renal cell carcinoma, according to her Leibovich scoring, with laboratory tests and imaging every six months for the first three years and then annually. An interval scan at 18 months revealed a significant lung nodule measuring 1.5 cm on CT scan. An FDG-PET CT was subsequently performed, which showed an SUVmax avidity of 16.8 in the lateral segment after two years of follow-up in February 2022, and it was FDG avid. A thoracoscopic resection of the solitary pulmonary nodule was performed, which confirmed metastatic renal cell carcinoma (RCC), clear cell type. After discussion in the National Multidisciplinary Uro-oncology meeting, the consensus was to initiate immunotherapy comprising Nivolumab and Ipilimumab. During follow-up, she presented to the clinic with hematuria at the two-year mark from diagnosis, at the end of 2022. On evaluation, she was found to have an enhancing left uretero-vesical junction mass measuring approximately 2.9 × 2.7 cm, extending to the most distal part of the ureter (Figure 2).

**Figure 3:**
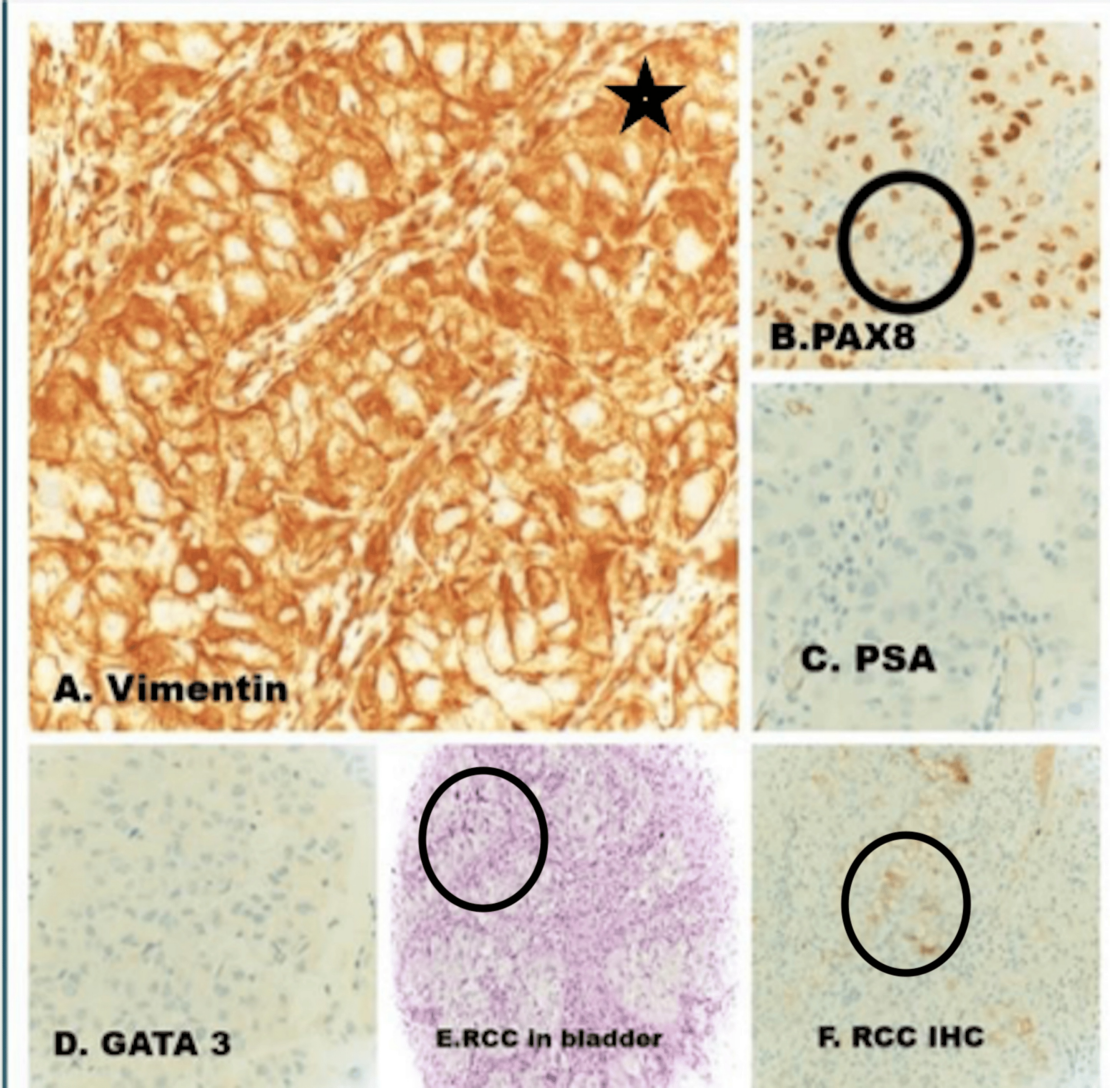
H&E section of the bladder TURB shows infiltration by nests of malignant polygonal cells with clear to pale eosinophilic cytoplasm, separated by a fine vascular network. (E, F) Immunohistochemistry supports the diagnosis of metastatic clear cell renal cell carcinoma, with positive staining in the tumor cells for RCC and vimentin (A), and PAX8 (B). Negative staining for PSA (C) rules out a prostate origin, and negative staining for GATA3 (D) rules out a bladder origin. H&E: hematoxylin and eosin, TURB: transurethral resection of bladder, RCC: renal cell carcinoma, PAX8: paired box gene 8, PSA: prostate-specific antigen, GATA3: GATA-binding protein 3

Cytology was negative for high-grade urothelial carcinoma. A stent was placed anterograde, and she underwent transurethral resection of a bladder tumor in December 2022. Histopathology revealed metastatic renal cell carcinoma, clear cell carcinoma subtype, and it was consistent with the previously excised tumor in 2020 (Figure 3).

The tumor invaded the muscularis propria (detrusor muscle). On immunohistochemistry, the lesional cells were positive for PAX8, CAIX, and vimentin. They were focally positive for CD10 and RCC, and negative for CK7, GATA3, and uroplakin. The morphological features and immunophenotype supported a metastatic renal cell carcinoma of the clear cell variant.

**Figure 4:**
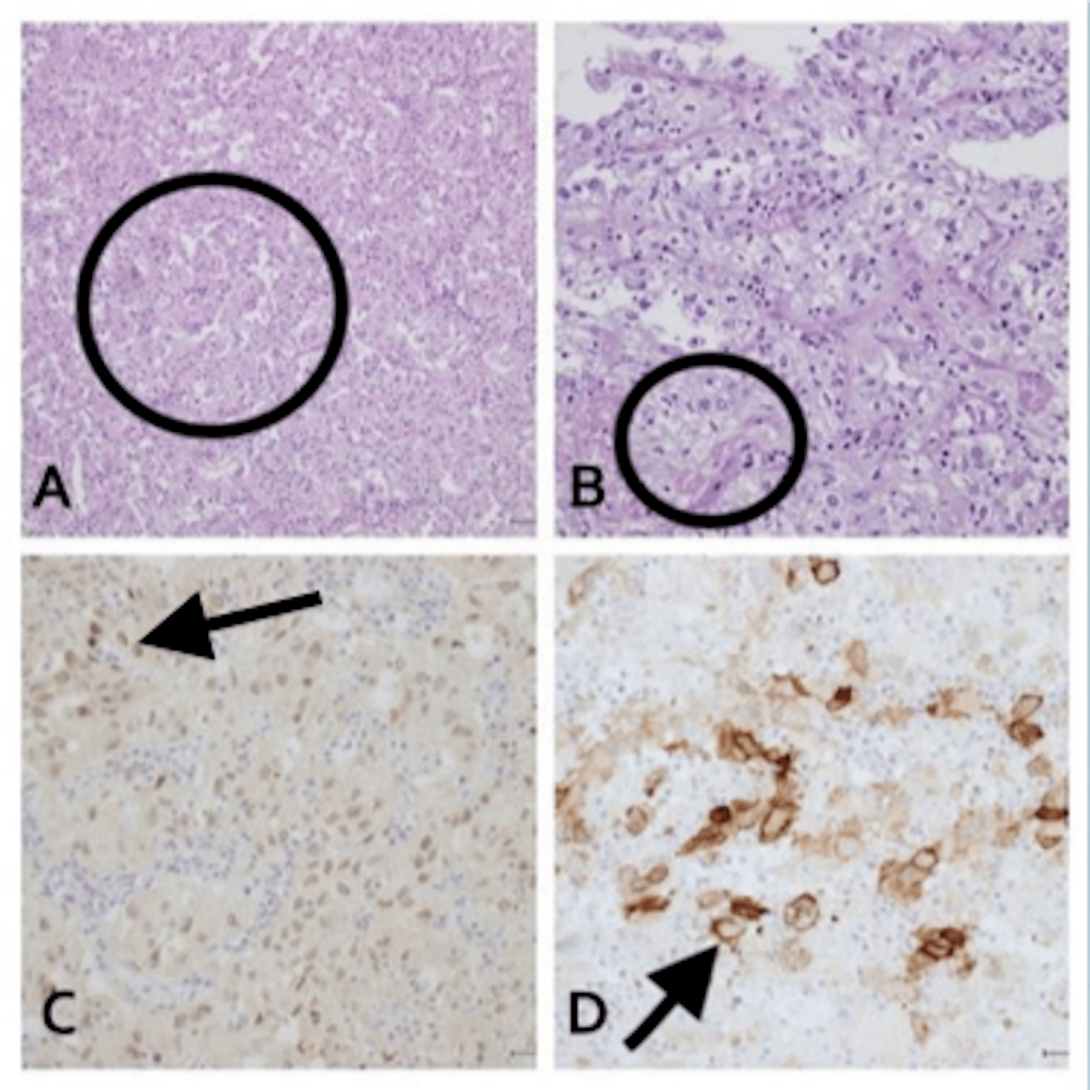
Cystectomy specimen shows high-grade tumor arranged in nests (A) with patchy necrosis. The tumor exhibits clear to eosinophilic cytoplasm, marked nuclear pleomorphism, and frequent mitotic figures (B). By immunohistochemical studies, the tumor is positive for PAX8 (C) and CD10 (D), confirming metastatic clear cell renal cell carcinoma.

After discussion in the national uro-oncology multidisciplinary team meeting, the consensus was to perform an anterior exenteration with an ileal conduit due to significant hematuria. The final histopathology confirmed metastatic clear cell renal cell carcinoma (Figure 4).

The patient is under regular follow-up in our hospital with medical oncology and urology. She has completed her course of immunotherapy, and the latest follow-up FDG PET/MRI study shows no newly developed hypermetabolic FDG-avid locoregional disease recurrence or distant metastasis.

## Discussion

Metastasis of RCC to the urinary tract is extremely rare. In 1907, Hoffmann described the first case of RCC metastasizing to the bladder [7]. Such metastasis can occur either synchronously or metachronously [8]. Typically, patients with this condition exhibit widespread tumor dissemination, including metastasis to other organs [9].

An earlier recurrence following nephrectomy has been linked to a higher tumor stage, with T3 tumors typically recurring within 17 to 28 months [10]. In the case discussed here, recurrence happened after two years and was uniquely presented in the bladder, an uncommon site for RCC recurrence. The exact pathologic mechanism for RCC spread to the urinary bladder is still not fully understood. Suggested mechanisms include hematogenous metastasis via the systemic circulation, retrograde tumor spread from the renal vein or renal hilar lymphatics to the periureteral veins or lymphatics connected to pelvic organs, and direct intraluminal migration of tumor cells leading to the seeding of the distal urothelium [8,11].

RCC primarily spreads through the bloodstream, often resulting in the simultaneous detection of widespread metastases. In contrast, urinary spread may be considered if the primary tumor invades the renal pelvis or collecting duct [12]. Despite evidence of hematogenous spread to other organs and invasion of the renal vein in our case, the bladder metastasis occurred in the opposite ureteric orifice, leaving the pathogenesis still elusive.

Treatment options for metastatic RCC are limited because chemotherapy and radiation therapy show poor effectiveness. However, the prognosis is favorable when there is a solitary bladder metastasis, enabling follow-up without the need for additional systemic therapy after surgical removal of the metastatic lesion [13]. When carcinoma has already metastasized to other organs by the time RCC is found in the bladder, additional systemic treatments, such as immunotherapy, are required. In our case, immune therapy was administered, and due to significant hematuria, anterior exenteration was performed, resulting in complete surgical resection of the metastatic tumor.

Clear cell carcinomas originating from other organs, including the prostate, lung, breast, uterus, ovary, and vagina, can rarely metastasize to the bladder. Additionally, metastatic melanoma, clear cell sarcoma, and seminoma are other potential diagnoses that should be taken into account [14]. In the present case, the differentials mentioned were ruled out based on morphology and immunohistochemistry.

## Conclusions

Metastasis of RCC to the bladder is exceptionally uncommon and can occur synchronously or metachronously. This potential occurrence should be taken into consideration when RCC patients experience hematuria following nephrectomy. Though uncommon, this possibility should always be considered when assessing clear cell tumors of the urinary bladder, regardless of whether the patient has a prior history of RCC.

## References

[1] Bukowski RM (1997). Natural history and therapy of metastatic renal cell carcinoma: the role of interleukin-2. Cancer.

[2] Padala SA, Barsouk A, Thandra KC (2020). Epidemiology of renal cell carcinoma. World J Oncol.

[3] Molina AM M D (2018). A multidisciplinary approach for the management of earlier stage renal cell carcinoma. Urol Oncol.

[4] International Agency for Research on Cancer (2004). Pathology and genetics of tumours of the urinary system and male genital organs. World Health Organization Classification of Tumours.

[5] Cronin RE, Kaehny WD, Miller PD, Stables DP, Gabow PA, Ostroy PR, Schrier RW (1976). Renal cell carcinoma: unusual systemic manifestations. Medicine (Baltimore).

[6] Zhang M, Wah C, Epstein JI (2014). Metastatic renal cell carcinoma to the urinary bladder: a report of 11 cases. Am J Surg Pathol.

[7] Hoffmann EH (1907). Nvpernephrom-metastasen. Dtsch Med Wochenschr.

[8] Gulati M, Gore JL, Pantuck AJ, Kim Y, Barajas L, Rajfer J (2007). Ureteral tumor thrombus from renal cell carcinoma extending into bladder. Urol Oncol.

[9] Sim SJ, Ro JY, Ordonez NG, Park YW, Kee KH, Ayala AG (1999). Metastatic renal cell carcinoma to the bladder: a clinicopathologic and immunohistochemical study. Mod Pathol.

[10] Chin AI, Lam JS, Figlin RA, Belldegrun AS (2006). Surveillance strategies for renal cell carcinoma patients following nephrectomy. Rev Urol.

[11] Raviv S, Eggener SE, Williams DH, Garnett JE, Pins MR, Smith ND (2002). Long-term survival after "drop metastases" of renal cell carcinoma to the bladder. Urology.

[12] Mayer WA, Resnick MJ, Canter D, Ramchandani P, Kutikov A, Harryhill JF (2009). Synchronous metastatic renal cell carcinoma to the genitourinary tract: two rare case reports and a review of the literature. Can J Urol.

[13] Uygur MC, Ozen HA, Sungur A, Remzi D (1994). A solitary and synchronous metastasis of renal cell carcinoma to the bladder. Int Urol Nephrol.

[14] Knez VM, Barrow W, Lucia MS, Wilson S, La Rosa FG (2014). Clear cell urothelial carcinoma of the urinary bladder: a case report and review of the literature. J Med Case Rep.

